# Supplementary material for: The Anti-Tumorigenic Role of Cannabinoid Receptor 2 in Colon Cancer: A Study in Mice and Humans
Source: Int J Mol Sci. 2023 Feb 17;24(4):4060. doi: 10.3390/ijms24044060 (PMC9961974; doi:10.3390/ijms24044060)
Supplement: Supplementary file 1 [file ijms-24-04060-s001.zip › ijms-2211646-supplementary.pdf]

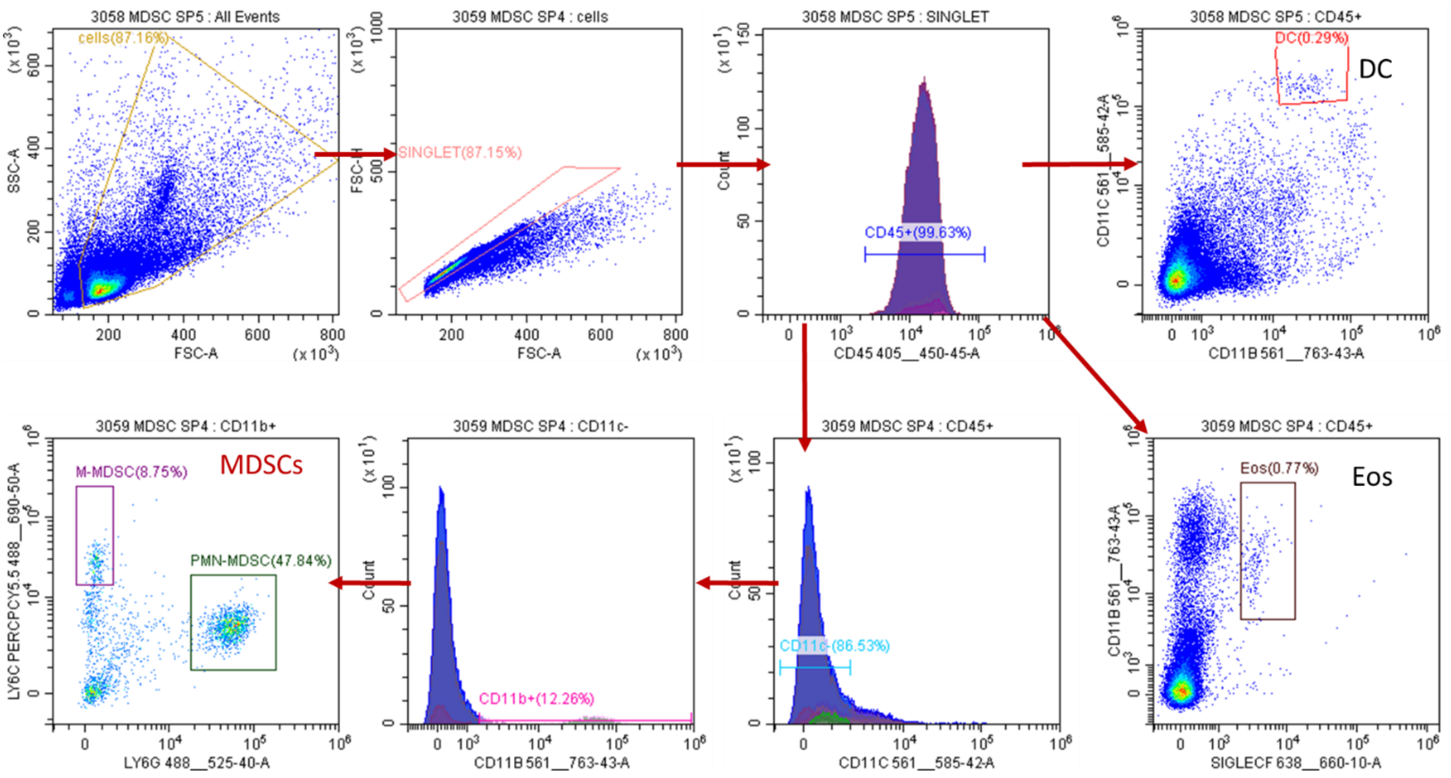

Supplementary Figure S1. Example Gating for myeloid cells in the spleen. Live cells were selected based on FSC and SSC, followed by singlet selection based on FSA-A and FSC-H. Dendritic cells were identified as CD45+CD11c^{hi}CD11b^{hi}. Eosinophils (Eos) were identified as CD45+CD11b+Siglec-F+. PMN-MDSCs were identified as CD45+CD11c-CD11b+Ly6G+Ly6C^{int}. M-MDSCs were identified as CD45+CD11c-CD11b+Ly6G-Ly6C-.

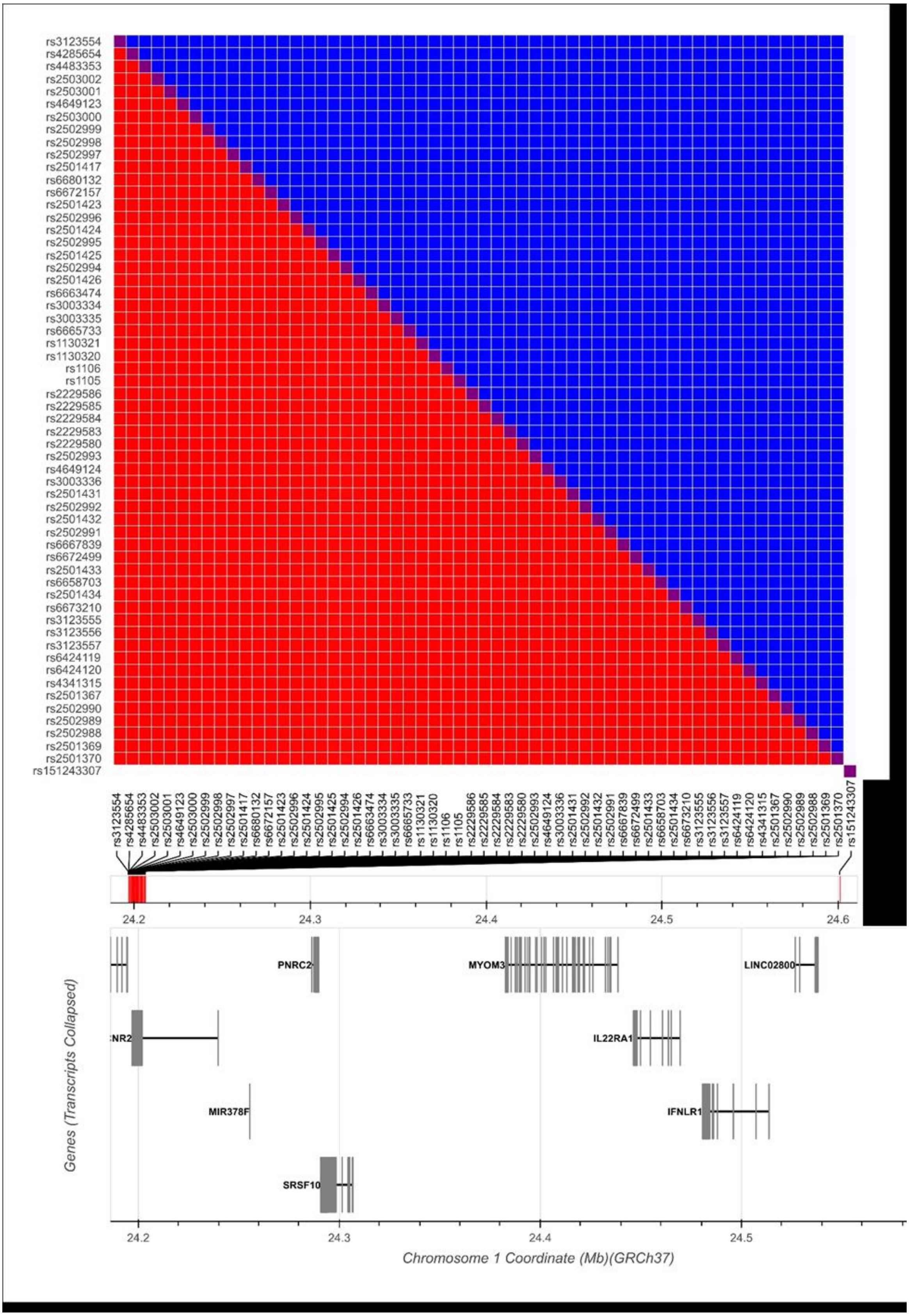

Supplementary Figure S2. LD Matrix encompassing the 63 significant SNPs found in the *CNR2* gene as well as the highly significant rs151243307 polymorphism that is external to the *CNR2* gene but belongs in the same haplotype block.
